# ^1^H, ^13^C and ^15^N NMR assignments of self-incompatibility protein homologue 15 from *Arabidopsis thaliana*

**DOI:** 10.1007/s12104-018-9853-0

**Published:** 2018-10-03

**Authors:** Rachel J. Coulthard, Karthik V. Rajasekar, Jon P. Ride, Eva I. Hyde, Lorna J. Smith

**Affiliations:** 1 Department of Chemistry, University of Oxford, Oxford, OX1 3QR UK; 2 Department of Biochemistry, University of Oxford, Oxford, OX1 3QU UK; 3 School of Biosciences, University of Birmingham, Birmingham, B15 2TT UK; 4 Present Address: EMBL Heidelberg, Meyerhofstraße 1, 69117 Heidelberg, Germany

**Keywords:** SPH proteins, PrsS, Self-incompatibility, Plant development

## Abstract

The SPH proteins are a large family of small, disulphide-bonded, secreted proteins, originally found to be involved in the self-incompatibility response in the field poppy (*Papaver rhoeas*). They are now known to be widely distributed in plants, many containing multiple members of this protein family. Apart from the PrsS proteins in *Papaver* the function of these proteins is unknown but they are thought to be involved in plant development and cell signalling. There has been no structural study of SPH proteins to date. Using the Origami strain of *E. coli*, we cloned and expressed one member of this family, SPH15 from *Arabidopsis thaliana*, as a folded thioredoxin-fusion protein, purified it from the cytosol, and cleaved it to obtain the secreted protein. We here report the assignment of the NMR spectra of SPH15, which contains 112 residues plus three N-terminal amino acids from the vector. The secondary structure propensity from TALOS^+^ shows that it contains eight beta strands and connecting loops. This is largely in agreement with predictions from the amino acid sequence, which show an additional C-terminal strand.

## Biological context

The S proteins of poppy, *Papaver rhoeas*, now called PrsS, were initially identified as the stigmatic component of the self-incompatibility response (Foote et al. 1994). Self-incompatibility is one of the most important processes in preventing self-pollination in plants. This occurs by different mechanisms in different plant species (Fujii et al. 2016). In *Papaver* the response involves S allele-specific recognition between the S proteins and a receptor in incompatible pollen, resulting in inhibition of pollen tube tip growth by apoptosis (Eaves et al. 2014). Several years after the discovery of the S proteins in *Papaver*, analysis of the genomic sequence of *Arabidopsis thaliana* revealed a set of genes with homology to the self-incompatibility alleles of *P. rhoeas* (Ride et al. 1999). As *A. thaliana* does not exhibit self-incompatibility and, given the taxonomic distance between *Papaver* and *Arabidopsis*, the discovery of these homologous genes in *Arabidopsis* and *Papaver* suggested that this gene family might be widespread within higher plants and have a larger role than just self-incompatibility. Since then genes for several SPH proteins (S protein homologues) have been found in most plants, even those with other mechanisms of self-incompatibility, with over 1400 sequences identified as belonging to the same Pfam group (PF05938) to date. There have been no structural studies of this group of proteins as yet, possibly because they contain disulphide bonds and are difficult to express in soluble form in bacteria.

Self-incompatibility Protein Homologue 15 (SPH15) is an 112 residue protein produced in *A. thaliana* (Ride et al. 1999). Its role in the physiology of *Arabidopsis* is currently unknown, although it has been suggested that the SPH family act as small, extracellular signalling proteins, possibly with involvement in plant development. To understand more about the structure and function of these proteins we have cloned and overexpressed SPH15 and begun to examine it by NMR spectroscopy. We here report the assignments of the ^1^H, ^15^N and ^13^C spectra of the protein and the secondary structure propensity from TALOS^+^ (Shen et al. 2009).

## Methods and experiments

### Cloning

The coding sequence of the SPH15 protein, minus the predicted signal peptide, and with a stop codon after the final amino acid, was cloned into pET32b (Novagen), using the *Nco*I and *Xho*I sites. Two bases (GC) were inserted after the *Nco*1 site to obtain the correct reading frame. After expression, purification and enterokinase cleavage, this gives the secreted SPH15 protein with three additional amino acids (AMG) at the N-terminus.

### Protein expression and purification

The Origami (λDE3) strain of *E. coli*, containing the plasmid pET32b-SPH15 expressing the Thioredoxin-His_6_-SPH15 fusion, was grown in M9 minimal media with ^15^N-NH_4_Cl and ^13^C_6_-glucose as the sole nitrogen and carbon sources, plus ampicillin, kanamycin and tetracycline, at 37 °C, to mid-log phase. It was induced with 1 mM IPTG and left at 37 °C for 30 min before reducing the temperature to 15 °C overnight. The culture was centrifuged and the cell pellets resuspended in buffer containing 20 mM Tris HCl, pH 7.5 (pH at room temperature), 100 mM NaCl, DNAse1 10 µg/ml; RNAse A 10 µg/ml, MgSO_4_ 10 mM, MnSO_4_, 10 mM, and PMSF 10 µg/ml. The cells were sonicated, centrifuged and the supernatant incubated with Ni-NTA His-bind resin (Merck), on ice, for 30 min. The resin was packed into a column and washed with ten column volumes of buffer containing 50 mM sodium phosphate, pH 8.0 and 300 mM NaCl, followed by a gradient of 0–150 mM imidazole in the same buffer, over ten column volumes. Fractions were monitored for the presence of the protein by Bradford assay and 15% SDS-PAGE.

The eluted protein was dialysed into buffer containing 20 mM Tris HCl pH 7.5, 100 mM NaCl, and 2 mM CaCl_2_, followed by cleavage with recombinant enterokinase, at room temperature for 2–3 days. The cleaved proteins were dialysed into buffer containing 20 mM sodium phosphate, pH 7.6, 100 mM NaCl, and 0.1 mM EDTA and loaded onto a Phosphocellulose, P11 column. The flow-through samples contained Thioredoxin-His_6_, while the SPH15 protein was eluted using a 100–600 mM NaCl gradient in the same buffer.

The SPH15 protein was concentrated by ultrafiltration with a 10 kDa cut-off filter and dialysed into buffer containing 20 mM sodium phosphate pH 5.2, 50 mM NaCl, and 0.1 mM EDTA for subsequent experiments.

#### NMR spectroscopy

2D and 3D CBCACONH, HNCACB, HNCO, HNCA, HNCACO, HBHACONH, HNHA, taken in H_2_O, and 3D-HCCH-COSY and 3D-HCCH-TOCSY spectra in D_2_O, (reviewed in Sattler et al. 1999), were acquired at 25 °C on a Bruker 500 MHz spectrometer with a TXI cryoprobe, using a 0.3 mM ^13^C/^15^N labelled protein sample. HCCONH and TOCSY-^15^N HSQC (in H_2_O) and HCACO spectra (in D_2_O) were acquired on a Varian 800 MHz spectrometer with a room temperature TXI probe using a ~ 1 mM protein sample.

Spectra were processed with NMRPipe (Delaglio et al. 1995) and analysed with NMR View 5 (Johnson and Blevins 1994) and CCPN (Vranken et al. 2005) software. Backbone resonances were assigned from triple resonance spectra and confirmed using HBHACONH, HNHA and HCACO spectra. The backbone assignments were extended to give side chain assignments using HCCH-COSY, HCCH-TOCSY, HC(C)(CO)NH-TOCSY and HC(C)NH-TOCSY spectra. ^1^H assignments for aromatic side chains were made using 2D ^1^H–^1^H TOCSY and ^1^H–^1^H NOESY spectra, taken on a Varian 600 MHz spectrometer.

## Extent of assignments and data deposition

**Fig. 1:**
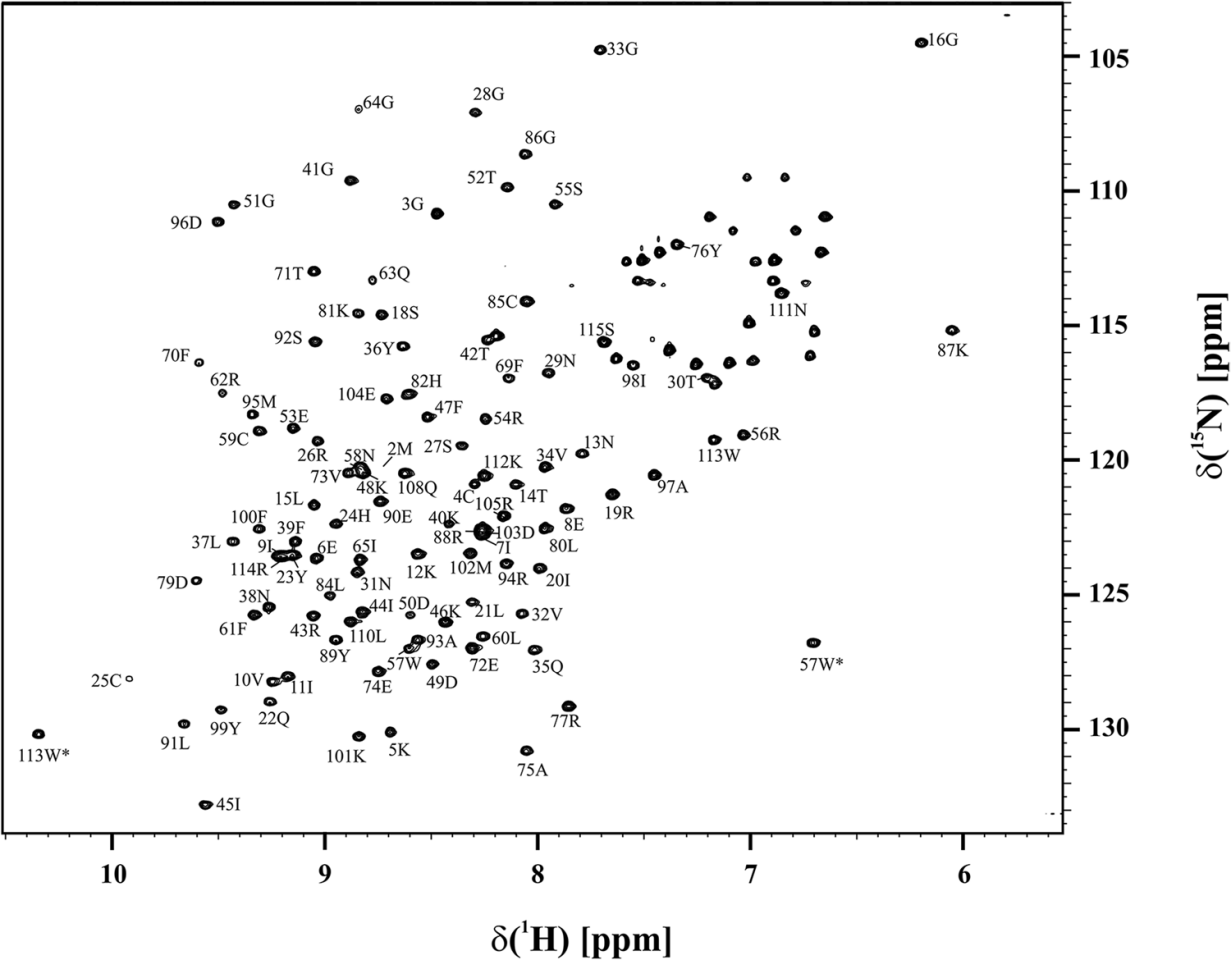
The ^1^H–^15^N HSQC NMR spectrum of SPH15, at 800 MHz, in 20 mM sodium phosphate buffer, pH 5.2, 50 mM NaCl, 0.1 mM EDTA. Assigned backbone NH cross-peaks are labelled with the corresponding residue numbers, with the amino acids from the vector as residues 1–3. The two peaks labelled with * are from the tryptophan indole NH groups

The ^1^H-^15^N HSQC spectrum of SPH15 is shown in Fig. 1. The spectrum is well-resolved with a single peak for each NH group, with 105 peptide amide peaks out of 108 expected, showing that the protein is folded with a single conformation. No NH peaks were observed for residues 66–68, which are presumably in a surface exposed loop. Assignments were made for all the backbone ^15^N_H_, ^13^C_α_, ^13^C_β_, ^1^H_N_, ^1^H_α_ and ^1^H_β_ resonances, apart from the ^13^C_β_ and ^1^H_β_ resonances of Glu 63, the ^15^N signals of the prolines, Ala 1, and Lys 68, and all the signals from residues 66, 67 and from Pro106, which is adjacent to another Proline residue. Side chain ^15^N and NH resonances were not assigned, other than the Trp indole NH groups. All but 10 ^13^C carbonyl resonances were assigned. ^13^C NMR resonance assignments were obtained for > 95% of the aliphatic side-chains; almost all the resonances apart from lysine δ and ε; proline γ, δ and ε resonances; Met εMe, and Arg guanidino groups. The associated ^1^H NMR resonances of these aliphatic side chains, and of 7 out of the 15 aromatic residues were also assigned.

**Fig. 2:**
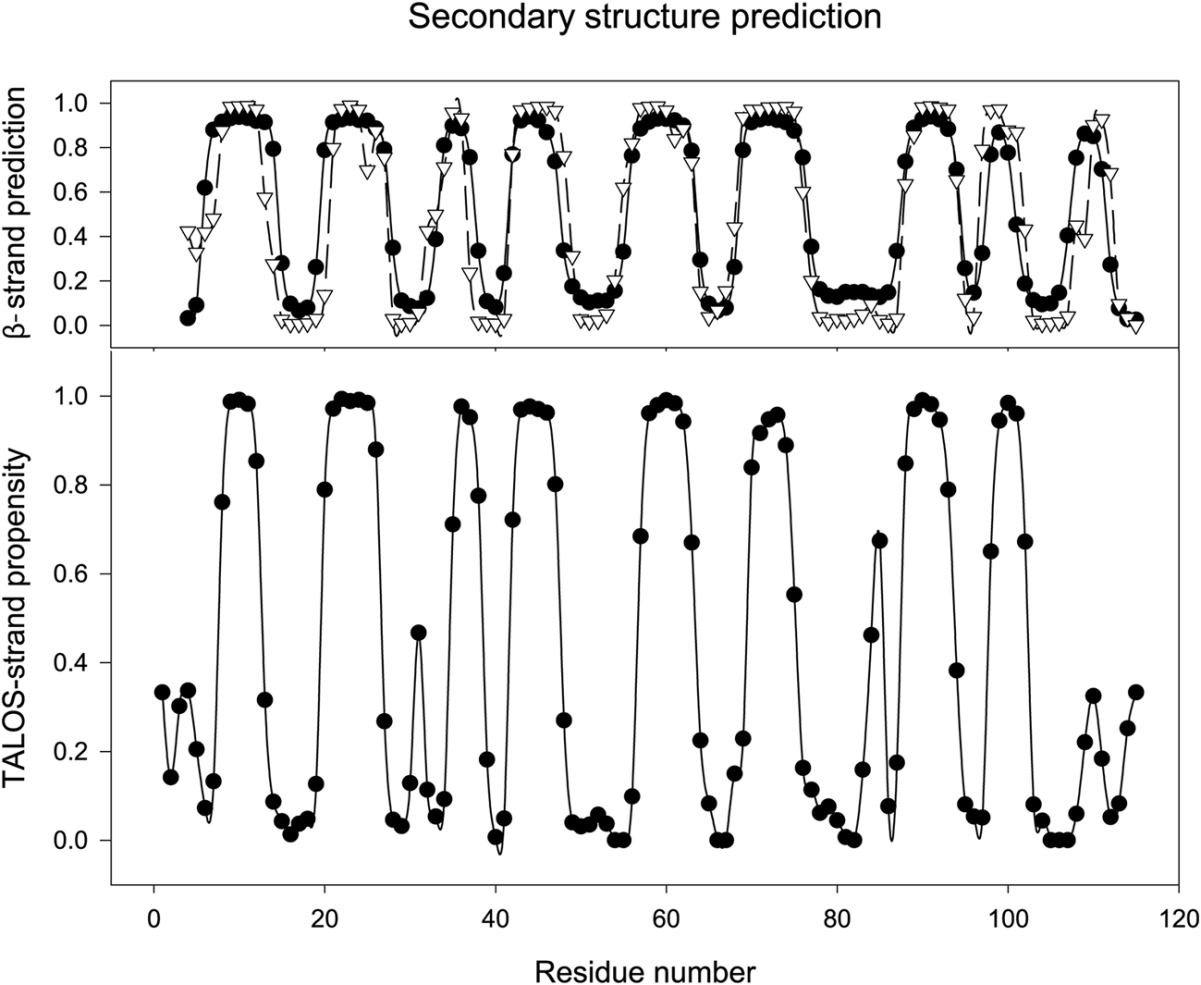
Secondary structure propensity predictions for SPH15. Top: prediction of extended, beta strand, conformation from the amino acid sequence: black circles, solid line-JPRED4 prediction (Drozdetskiy et al. 2015), white triangles and dashed lines-PSIPRED prediction (Buchan et al. 2013). Bottom: extended, beta strand, structure propensity from TALOS^+^ (Shen et al. 2009). Residues are numbered as in Fig. 1, including the residues from the vector as numbers 1–3

The secondary structure predictions for SPH15 and other members of the SPH protein family from the bioinformatics servers JPRED4 (Drozdetskiy et al. 2015), and PSI-PRED (Buchan et al. 2013) show no helical regions, but nine beta-strands (Fig. 2, top). Analysis of the intraresidue dihedral angles by TALOS^+^ (Shen et al. 2009) indicates only eight regions of beta-strand structure (Fig. 2, bottom), with the rest mainly as loops. The only stretch of residues for which TALOS^+^ shows a helical propensity > 50% is from amino acid 53–55, but, of these, only residue 54 is assigned to helix with a confidence level > 50% and such a short helix is unlikely. The eight regions of beta strand from TALOS^+^ are similar in position to those predicted from the servers, but a little shorter and the final C-terminal strand predicted is not seen in the NMR data. This final strand predicted by PsiPred and JPRED4 contains only 3 or 4 residues respectively, with the latter having Pro 109, which cannot form H-bonds, as the second residue. The C-terminus of a protein is often highly mobile, and, if this short strand is on the outside of the protein, it is likely to be thermally unstable and so not form under the NMR conditions.

The determination of the molecular structure using dihedral angles from TALOS^+^ and analysis of ^13^C- and ^15^N-edited NOESY is underway. The chemical shifts have been deposited in the BioMagResBank (http://www.bmrb.wisc.edu/) under the accession number BMRB-27459.
